# Soil phosphorus functional fractions and tree tissue nutrient concentrations influenced by stand density in subtropical Chinese fir plantation forests

**DOI:** 10.1371/journal.pone.0186905

**Published:** 2017-10-26

**Authors:** Xiang-Min Fang, Xiu-Lan Zhang, Ying-Ying Zong, Yang Zhang, Song-Ze Wan, Wen-Sheng Bu, Fu-Sheng Chen

**Affiliations:** 1 Jiangxi Provincial Key Laboratory of Silviculture, College of Forestry, Jiangxi Agricultural University, Nanchang, China; 2 Jiulianshan National Observation and Research Station of Chinese Forest Ecosystem, 2011 Collaborative Innovation Center of Jiangxi Typical Trees Cultivation and Utilization, Jiangxi Agricultural University, Nanchang, China; RMIT University, AUSTRALIA

## Abstract

Stand density regulation is an important measure of plantation forest management, and phosphorus (P) is often the limiting factor of tree productivity, especially in the subtropics and tropics. However, the stand density influence on ecosystem P cycling is unclear in Chinese fir (*Cunninghamia lanceolata*) plantations of subtropical China. We collected rhizosphere and bulk soils, leaves and twigs with different ages and roots with different orders to measure P and nitrogen (N) variables in Chinese fir plantations with low density (LDCF) and high density (HDCF) at Fujian and Hunan provinces of subtropical China. Rhizosphere soil labile P, slow P, occluded P and extractable P were higher in LDCF than HDCF at two sites. Meanwhile, P and N concentrations of 1-year-old leaves and twigs were higher in LDCF than HDCF and leaf N/P ratio generally increased with increasing leaf age at two sites. Rhizosphere *vs*. bulk soil labile P and occluded P were greater in LDCF than HDCF at Fujian. Nitrogen resorption efficiencies (NRE) of leaves and twigs were higher in LDCF than HDCF at Fujian, while their P resorption efficiencies (PRE) were not different between two densities at two sites. The average NRE of leaves (41.7%) and twigs (65.6%) were lower than the corresponding PRE (67.8% and 78.0%, respectively). Our results suggest that reducing stem density in Chinese fir plantations might be helpful to increase soil active P supplies and meet tree nutrient requirements.

## Introduction

Stand density regulation and its ecological effects represent important measures of forest management that have been widely studied [1,2]. Decrease in stand density usually reduces the competition for soil nutrients, such as phosphorus (P) and nitrogen (N), between trees and promotes the growth of remaining plants [3]. Meanwhile, the variation in light, water, temperature and forest microenvironments prompted by stand density directly affects plant growth [4]. Moreover, the nutrient status of a tree may be indirectly influenced by stand density, since the litterfall biomass [5], litter decomposition rate, photosynthate distribution and other ecological processes that influence soil nutrient supply vary with the stand density [6,7]. Currently, the majority of studies of stand density have focused on its effect on tree radial growth and understory plant diversity [8,9], but the precise role of stand density in soil nutrient supply and plant nutrient distribution remains to be determined.

Soil P is one of the most important elements limiting plant growth, especially in subtropical and tropical regions [10,11]. In natural systems, soil P mainly originates from the slow weathering of soil minerals with low inputs to soil, coupled with the adsorption and chemical fixation on clay and other soil components, such as amorphous iron and aluminum, which results in a low P availability and increases the limitation of P on plant growth [12,13]. In soil solution, P exists in a variety of forms with different ecological functions, but only a small portion of the inorganic P can be directly absorbed by plants [14]. Therefore, accurate estimate of the concentration and change of P forms is critical for understanding the ecological functions of soil P [15]. The Hedley fractionation method recognizes plant-available forms and refractory forms of soil P and has been widely used in research into the P dynamics of natural and managed ecosystems [16,17,18]. Although the impact of stand density on soil P dynamics has previously been investigated [19], the response of soil P functional fractions associated with plant availability is not yet fully understood.

The rhizosphere encompasses the millimeters of soil surrounding a plant root and soil microbes, invertebrates and root systems of competitors [20]. In the rhizosphere, the amounts of root exudates and soil microbes are strongly different from the bulk soil, mediating the biological interactions and ecological processes, which is known as the rhizosphere effect [20,21]. The rhizosphere contains more micro-organisms feeded by the organic substrates from the root. In turn, these micro-organisms can influence the plant as primary producer via decomposing organic matter and releasing inorganic nutrients [20]. Thus, the rhizosphere effect on soil nutrients has been successfully applied to assess nutrient limitation and has the potential to be an indicator of plant nutrient requirements [22], since plants may develop an enhanced physiological capacity to extract nutrients from soil through increasing rhizosphere effect when they encounter infertile soils [21,22]. Accordingly, plants may adjust the strength of the rhizosphere effect in response to relative nutrient deficiency in a denser stand. Moreover, as another conservation strategy for adapting nutrient deficiency, resorption is considered as a key process in internal, whole-plant nutrient cycling [23,24], defined by the withdrawal of nutrients from senescing organs and the subsequent transportation to storage sites and growing organs. Nutrient resorption in rapid turnover organs ensures that nutrients are available for future growth and reduces the dependence on external nutrient availability, especially in ecosystems with poor nutrient status [11]. Plants usually exhibit different nutrient resorption strategies in response to the dissimilar nutrients status induced by stand density [25]. Consequently, the study of internal nutrient cycling within an ecosystem including soil rhizosphere processes and plant nutrient accumulation and resorption, is required for a broader understanding of the responses of forest ecosystems to different stand densities.

Chinese fir (*Cunninghamia lanceolata* (Lamb.) Hook.) has been one of the most important plantation tree species in the subtropics [26]. The planting area has reached 8.54×10^6^ ha and accounts for 21.4% of all plantation land in China. In many Chinese fir plantations, primary production and other basic ecosystem processes are constrained by low rates of nutrient supply, particularly by P availability. As a “rock-derived” essential element, P availability is often lower in old, highly weathered and strongly leached soils in the tropics and subtropics [10,26]. In recent years, excessive stand density in Chinese fir plantations has been considered a major problem that results in the decline of soil fertility and threatens sustainable stand productivity [2]. However, little is known about whether decreases in stand density can increase soil P supply and tree tissue P, and subsequently improve the internal P cycling in Chinese fir plantations.

In this study, we assessed the potential effect of stand density on nutrient internal cycling by sampling rhizosphere and bulk soils to examine the components of P functional fractions. Meanwhile, we collected main tissues (including three functional roots, three age-class leaves and twigs) from trees to measure P and N concentrations in low density and high density Chinese fir plantations (LDCF and HDCF, respectively) at two sites in subtropical China. We also calculated the rhizosphere effect of soil P supply and nutrient resorption efficiency. Our main hypotheses are: (1) Soil available P, tree tissue P and N concentrations are higher in LDCF than HDCF, given that the nutrient condition is improved in plantations with reduced tree competition and (2) The belowground P rhizosphere effect and aboveground P resorption efficiency are greater in HDCF than LDCF because of the stronger nutrient limitation under a higher stand density. These results could provide important information for stand density regulation of forest management in the Chinese fir plantations.

## Materials and methods

### Study area

The study was conducted at two sites of subtropic China. One study site was located in Datian country of Fujian Province (25°45′N, 117°33′E) and the other site was located in Huitong country of Hunan Province (26°52′N, 109°42′E). The two sites are about one thousand kilometers apart, but have the similar mid-subtropical, seasonal damp monsoon climate with a mean annual rainfall of approximately 1400 mm, which mainly occurs between March and September. The mean air temperature ranges from 15°C to 19°C and mean annual frost-free period is about 280–290 d yr^−1^. The soil is described as red Humic Planosol (FAO) developed from an acidic sandy shale, which is moderately well drained and clay textured [2]. The zonal vegetation of two sites was originally evergreen broadleaved forest, which has been nearly completely removed by human activities. Chinese fir plantation has become the major forest type because of its excellent economic benefits. The research site did not involve endangered or protected species, and no specific permissions were required for conducting experiment in this site.

### Experimental design

In May 2013, two Chinese fir stand types of low density (LDCF) and high density (HDCF) were selected at the two sites. In each site, four 200-m^2^ circular replicated plots were randomly chosen in different separating hills for each density plantation, and the plots were generally located at middle slope locations and were at least 250 m away from adjacent plots.

At Fujian, the LDCF and HDCF stands were established from 1989 to 1991. Before Chinese fir planting, this aera was dominated by evergreen broadleaved tree species, such as *Castanopsis fargesii* Franch., *Schima superba* Gardn. et Champ., *Cryptocarya chingii* Cheng, *Castanopsis faberi* Hance, *Castanopsis eyrei* (Champ.) Tutch., *Castanopsis tibetana* Hance and so on. During 1989–1991, evergreen broadleaved forests were removed and Chinese fir was planted since it was a fast-growing tree with high yield and marketable wood quality. The initial planting density was about 2700 trees per hectare. Similarly, at Hunan, the LDCF and HDCF stands were established in the eraly of 1983 after the evergreen broadleaved forests logging. The initial planting density was about 1800 trees per hectare. Two stand types of the two sites with different densities were formed in the seventh or eighth year with different thinning intensities. In this study, the average stand densities in LDCF and HDCF were about 1400 stems ha^−1^ and 2510 stems ha^−1^ at Fujian, and were about 1187 stems ha^−1^ and 1475 stems ha^−1^ at Hunan, respectively. Other stand characteristics are shown in Table 1. The two stand types were similarly managed; for example, weeds were controlled during the first 3 years and the stands were thinned and fertilized once during the first 10 years. In the fourth year of planting, the fertilizer was applied about 200 kg per hectare using groove fertilization method, in which the percentages of N, P_2_O_5_ and K_2_O were 17%, 7% and 6% respectively. The thinning effects, such as residues disposal, on the nutrient distribution were not considered, since it was done once more than ten years ago.

**Table 1:** Stand characteristics in low density and high density Chinese fir plantations (LDCF vs. HDCF) at two sites of subtropical China.

	Density (stems ha^−1^)	DBH (cm)	Height (m)	Fern cover (%)	Herb cover (%)
Fujian					
LDCF	1400±94b	20.9±1.2a	15.6±0.8	82.4±4.0	12.6±2.3
HDCF	2510±128a	18.2±0.2b	16.9±0.4	80.8±5.3	8.6±4.0
Hunan					
LDCF	1187±65b	25.32±1.0a	16.9±0.6a	9.0±3.0	40.9±12.8
HDCF	1475±90a	17.86±0.2b	10.1±0.5b	4.4±0.9	27.4±12.4

Value = Mean±1 standard error. DBH = diameter at breast height. Different letters indicate significant differences between LDCF and HDCF at 0.05 level.

### Soil and plant sampling

In May 2013, four representative trees were selected on the basis of the average DBH and height in each plot. For each sample tree, we used a steel corer to obtain three 15 cm × 15 cm× 15 cm soil blocks at a distance of 1.5 m from the trunk and divided soil samples into rhizosphere soil and bulk soil using the shaking method [21]. The soil adhered to the root surface within 2 mm after shaking was considered the rhizosphere soil, while the remaining soil after rhizosphere soil collection was considered bulk soil. The rhizosphere and bulk soil in each plot were completely mixed to obtain respective composite samples. All soil samples were brought to the laboratory for pretreatment. After roots and organic debris were removed by hand, the soil samples were air-dried, passed through a 2-mm sieve for pH analysis, and ground to pass through a 0.15-mm sieve for the determination of organic carbon (C), total N, total P and P fractions.

Plant sampling was carried out simultaneously with soil sample collection. In the middle and upper of tree canopy, twigs and leaves from one first-order branch were collected from the four directions of each representative tree to decrease heterogeneity induced by illumination. Twigs and leaves were divided into 1-year (the first order of branching), 2-year (the second order of branching) and 3-year (the third order of branching) tissues [11]. Twig and leaf samples of the same order in each plot were mixed completely to obtain a composite sample. Twig and leaf litter were also collected using three 1-m^2^ mesh (0.025 cm) litter boxes in each plot under the representative trees but were not differentiated by age. During the collection of rhizosphere soil, root samples with a diameter of less than 1 cm were collected and transported to laboratory. After washing with deionized water, we removed other plant roots and dead Chinese fir roots, and divided living Chinese fir roots into three functional roots including absorption, transportation, and storage roots, according to their order positions in the root system [27] Briefly, root segments with zero, one and two orders of visible dependent laterals were considered to be first, second and third order roots, respectively, and so on. The first to third order roots were mixed and were defined as absorption root. Similarly, the fourth and fifth order roots were defined as transportation roots. It was considered as storage root if the root orders were more than fifth and the diameter was less than 8 mm. All the plant samples were oven-dried, ground and screened with a 0.25-mm sieve prior to total N and P analysis.

### Chemical analysis of soil and plant samples

Soil pH (soil:water = 1:2.5) was determined with a PHS-3C pH meter (Shanghai Lida Instrument Factory, China). Soil organic C was determined by the Walkley–Black wet oxidation method following the removal of carbonates by acid pretreatment [28]. Total N and total P in both soils and tree tissues were determined by the Kjeldahl method and by the molybdenum-antimony colorimetry method, respectively, after the samples were digested with H_2_SO_4_ [28]. Additionally, the stoichiometric ratios of C, N and P were calculated based on their concentrations.

Soil P fraction was obtained with a sequential extraction procedure using the improved Hedley P fractionation method [12,29]. Air-dried soils (2 g) were shaken in centrifuge tubes with distilled water with 1g resin, 0.05 M NaHCO_3_, 0.1 M NaOH, 0.1 M NaOH with sonication, 1 M HCl and H_2_SO_4_-H_2_O_2_ digestion, respectively. The P concentrations in supernates extracted by the chemical solutions mentioned above were determined using the phosphomolybdic acid blue color method and respectively referred to as the concentrations of resin-P, NaHCO_3_-P, NaOH-P, sonication-P, HCl-P and residue-P, which were defined as available P, labile P, slow P, occluded P, weathered mineral P and inert P, respectively [18,30]. Extractable P includes available P, labile P, slow P, occluded P and weathered mineral P.

### Rhizosphere effect and nutrient resorption efficiency

Rhizosphere effects of soil P fractions were calculated using the ratio of P concentration in rhizosphere soil (R) to bulk soil (S). Here, R/S> 1 indicates a positive rhizosphere effect and R/S <1 indicates a negative rhizosphere effect [27].

Nutrient resorption efficiency is the percentage of the difference between the living and litter organ (such as leaves and twigs) nutrients divided by living organs nutrient concentration [11,23]. Nutrient resorption efficiency was calculated using the formula of Aerts (1996) as follows [23]: nutrient resorption efficiency = [(nutrient in living leaf or twig tissue − nutrient in the litter component corresponding to the same organs)/(nutrient in living leaf or twig tissue)] × 100%. In the present study, we divided living leaves and twigs into three age-classes but we did not identify the ages of leaf and twig litter. Thus, 1-year-old living leaf and twig were used as the living organ to obtain the maximum nutrient resorption efficiency, since 1-year-old leaf and twig usually have higher nutrient concentrations than those of older leaves and twigs [11].

### Statistical analysis

All statistical analyses were conducted using SPSS 16.0 [31]. All data were tested for homogeneity of variance (Levene’s test) before statistical analysis. Student’s *t*-test analysis was used to compare the differences of organic C, total N, total P and P fractions between LDCF and HDCF. The LSD multiple comparisons method was used to identify significant differences of total N, total P and N/P ratio in tree tissues among different functional groups or ages following ANOVA tests for significance. Two-way ANOVA was used to examine the effects of stand density, study site and their interactions on soil and plant nutrient properties. Pearson’s correlation analysis was performed to examine relationships between rhizosphere soil P fractions and tree tissue nutrient concentrations. The standard 0.05 level was used throughout as the threshold for statistical significance.

## Results

### Soil general characteristics

Rhizosphere soil organic C and total N concentrations were higher in LDCF than HDCF at two sites except total N at Fujian, however, pH and C/N were not significantly different between LDCF and HDCF at two sites (Table 2). Furthermore, there was no significant difference in bulk soil pH, total N and C/N between the two stand densities at two sites, while bulk soil organic C was higher in LDCF than HDCF at Hunan. Stand density had signifianct effects on rhizosphere and bulk soil organic C as well as bulk soil C/N. Meanwhile, study site had signifianct effects on soil organic C and total N in rhizosphere and bulk soil, respectively. No interactions of stand density and study site on soil general characteristics mentioned above were found (Table 2).

**Table 2:** Physiochemical properties in rhizosphere and bulk soils in low density and high density Chinese fir plantations (LDCF vs. HDCF) at two sites of subtropical China.

	pH		OC		TN		C/N	
	RS	BS	RS	BS	RS	BS	RS	BS
Fujian								
LDCF	4.31±0.04	4.23±0.04	31.13±3.02a	24.78±1.70	0.95±0.12	0.82±0.10	33.36±1.53	30.90±1.98
HDCF	4.35±0.03	4.33±0.01	21.68±1.07b	18.85±2.04	0.75±0.08	0.56±0.05	30.72±5.30	33.74±2.04
Hunan								
LDCF	4.36±0.20	4.36±0.21	28.07±2.71a	14.84±1.75a	1.09±0.07a	0.69±0.04	25.62±1.05	21.28±1.52
HDCF	4.09±0.06	4.03±0.03	15.99±0.96b	11.33±1.99b	0.58±0.02b	0.50±0.01	27.60±1.04	19.06±1.54
Variance analysis of *F*-statistics^#^								
SD	0.98^NS^	0.59^NS^	4.73*	21.69***	0.02^NS^	2.42^NS^	3.62^NS^	46.25***
SS	1.17^NS^	1.05^NS^	25.02***	6.34*	19.06***	15.21**	0.01^NS^	0.03^NS^
SD×SS	2.19^NS^	4.10^NS^	0.37^NS^	0.42^NS^	3.55^NS^	0.33^NS^	0.66^NS^	2.01^NS^

Value = Mean±1 standard error. Different letters indicate significant differences between LDCF and HDCF at 0.05 level. OC: organic carbon. TN: total nitrogen. RS: rhizosphere soil. BS: bulk soil. SD: stand density. SS: study site.

^#^ Significance level of *F* values

^NS^ not significant

* *p* < 0.05

** *p* < 0.01

*** *p* < 0.001.

### Total P and P fractions in rhizosphere and bulk soils

**Fig 1:**
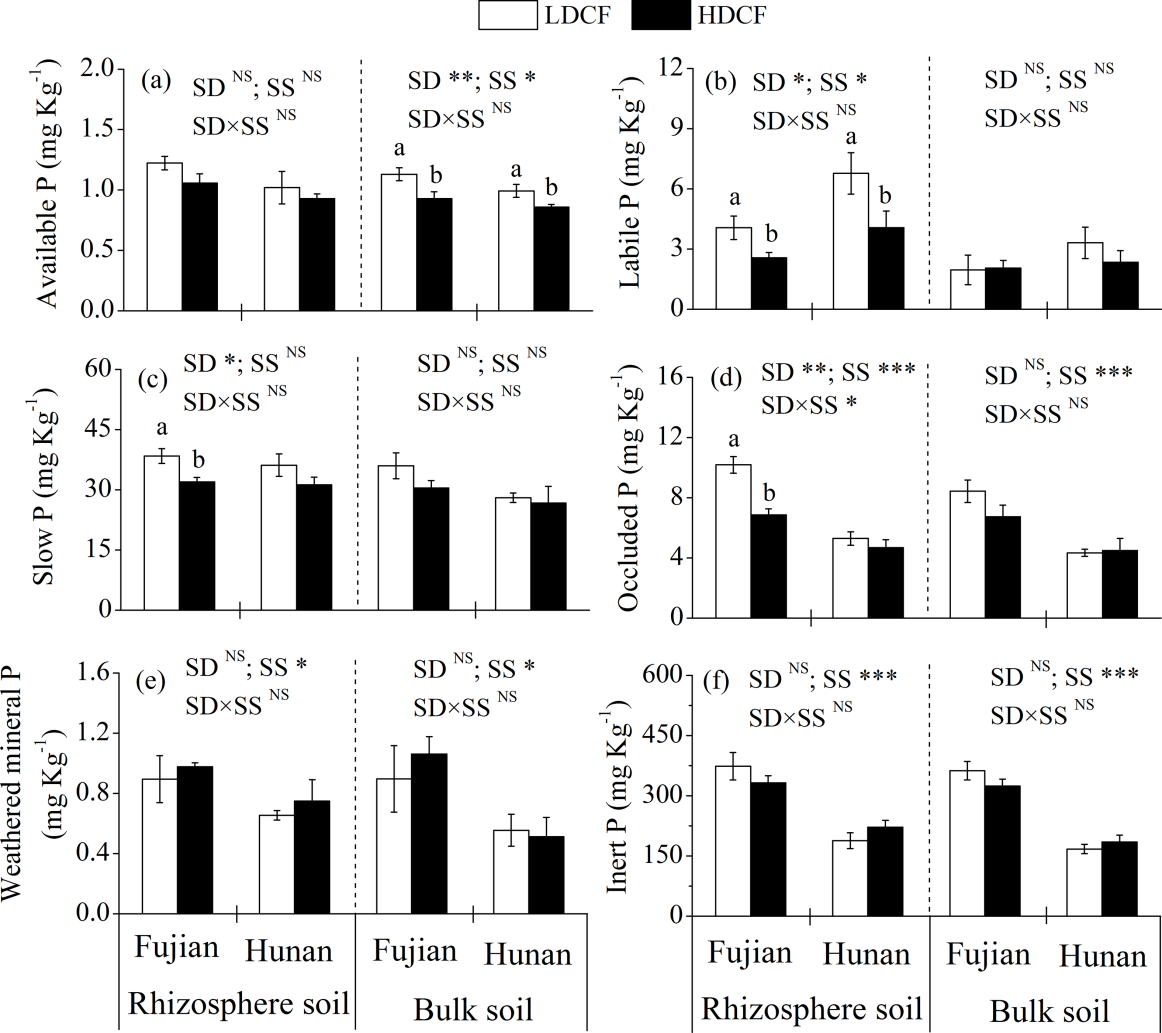
Phosphorus fractions in rhizosphere and bulk soils in low density and high density Chinese fir plantations (LDCF vs. HDCF) at two sites of subtropical China. Note: Mean±1 standard error. Different letters indicate significant differences between LDCF and HDCF in rhizosphere or bulk soil at the 0.05 level. SD: stand density. SS: study site. ^NS^ not significant, * *p* < 0.05, ** *p* < 0.01, *** *p* < 0.001.

There was a significant effect of stand density on all rhizosphere soil P fractions except for available P, weathered mineral P and inert P at two sites (Fig 1). At Fujian, the concentrations of rhizosphere soil labile P, slow P and occluded P were higher by 58.4%, 20.2% and 48.5%, respectively, in LDCF than HDCF. Similarly, the concentration of rhizosphere soil labile P was higher in LDCF than HDCF at Hunan (Fig 1B). Bulk soil available P was significantly higher in LDCF than HDCF at two sites, while no significant differences were detected for other bulk soil P fractions (Fig 1).

**Fig 2:**
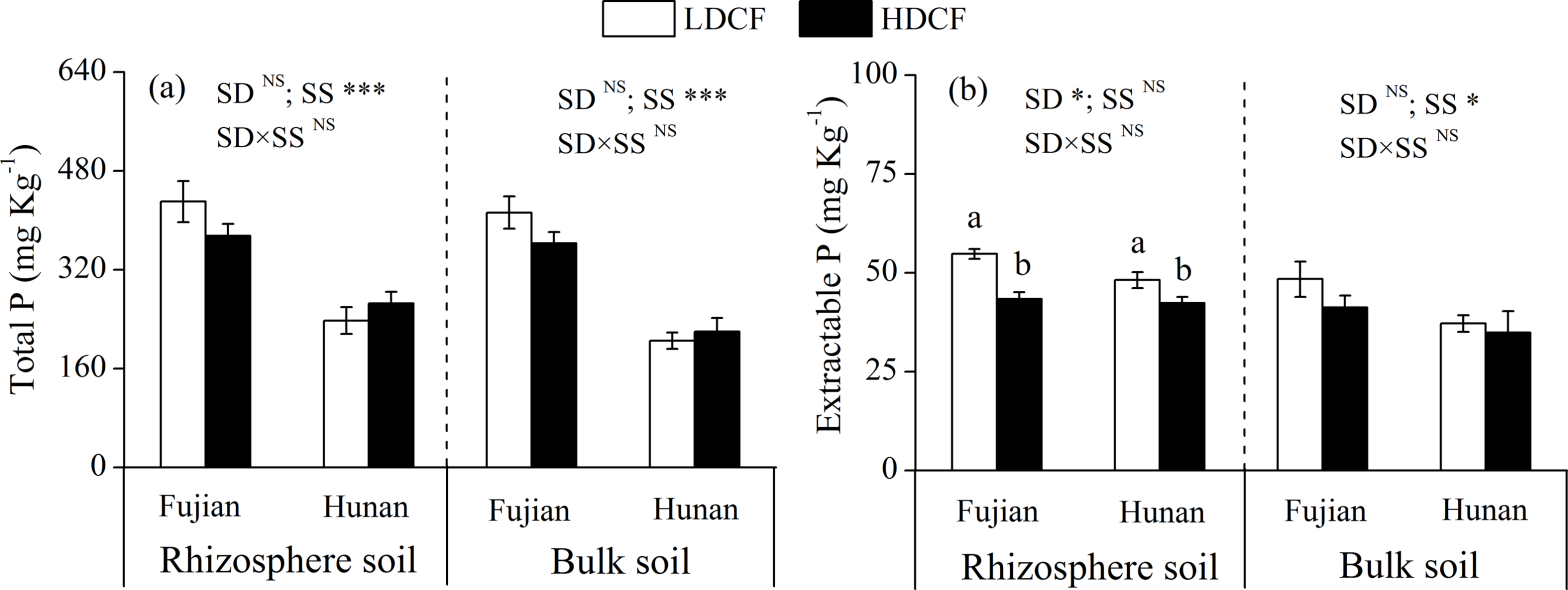
Total phosphorus and extractable phosphorus in rhizosphere and bulk soils in low density and high density Chinese fir plantations (LDCF vs. HDCF) at two sites of subtropical China. Note: Mean±1 standard error. Different letters indicate significant differences between LDCF and HDCF in rhizosphere or bulk soil at the 0.05 level. SD: stand density. SS: study site. ^NS^ not significant, * *p* < 0.05, ** *p* < 0.01, *** *p* < 0.001.

Soil total P was not significantly different between LDCF and HDCF, while the rhizosphere extractable P was higher in LDCF than HDCF at two sites (Fig 2). In addition, the average soil total extractable P concentrations in LDCF and HDCF accounted for 12.2% and 11.5% of total P at Fujian and accounted for 19.0% and 16.3% of total P at Hunan, respectively. No interactions of stand density and study site on soil total P, extractable P and P fractions were found except for rhizosphere soil occluded P (Figs 1 and 2).

### Nutrients in leaves, twigs and roots

**Fig 3:**
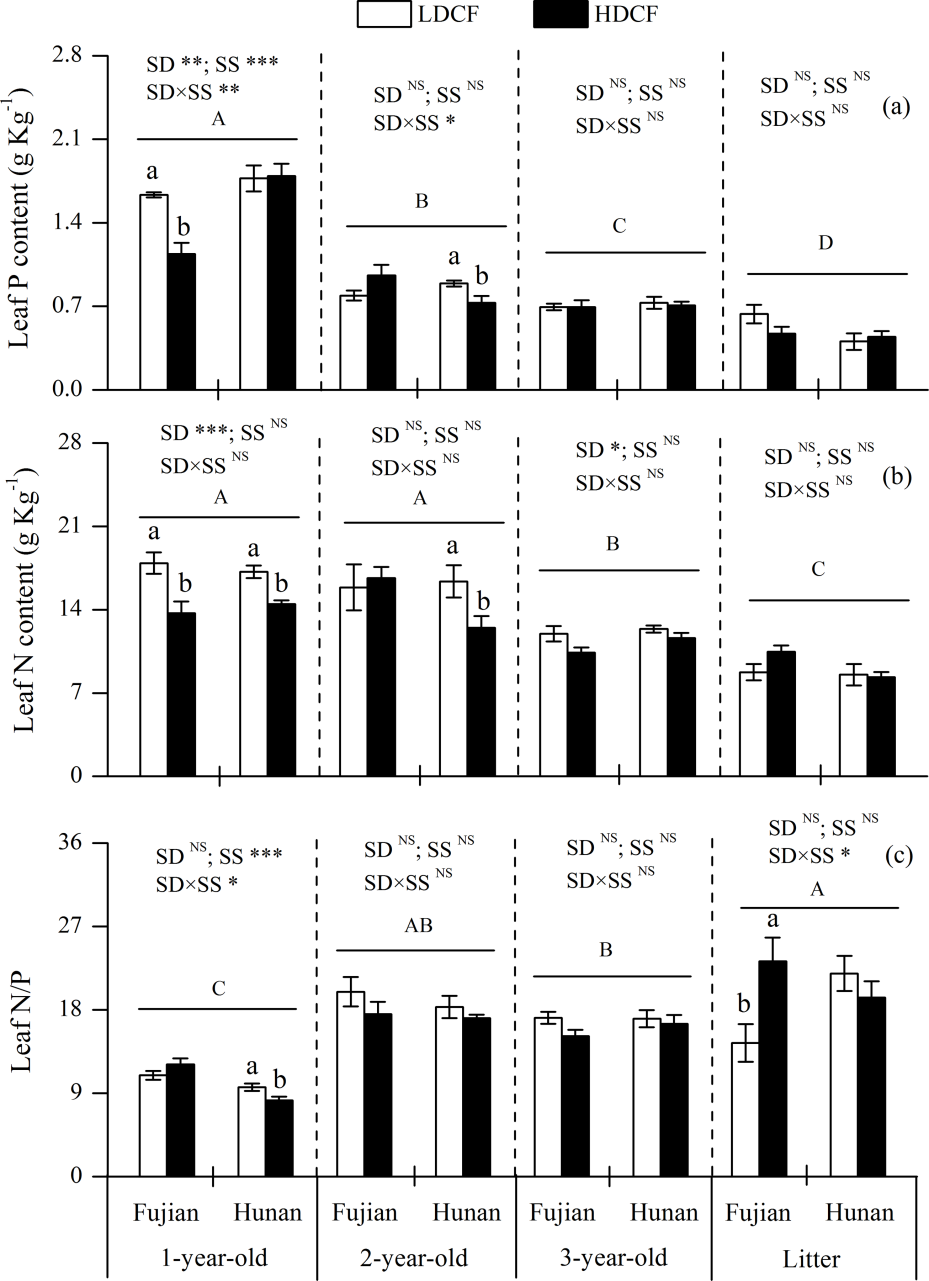
Nitrogen and phosphorus concentrations and their stoichiometry in leaves with different ages in low density and high density Chinese fir plantation (LDCF vs. HDCF) at two sites of subtropical China. Note: Mean±1 standard error. Different small letters indicate significant differences between LDCF and HDCF in leaves of the same age at the 0.05 level. Different capital letters indicate significant differences among leaves of different ages at the 0.05 level. SD: stand density. SS: study site. ^NS^ not significant, * *p* < 0.05, ** *p* < 0.01, *** *p* < 0.001.

The P concentration was higher by 43.4% of 1-year-old leaves at Fujian and higher by 22.3% of 2-year-old leaves at Hunan in LDCF than HDCF. Meanwhile, 1- year-old leaves N concentration was higher in LDCF than HDCF at two sites. Stand density had a significant effect on 1-year-old leaf P and N concentrations (Fig 3A and 3B), however, there were no significant differences in P and N concentrations of 3-year-old leaves and leaf litters between two stand densities. The leaf P and N concentrations generally decreased with leaf age. The P concentration was the highest in 1-year-old leaves and the N concentrations in 1-year-old and 2-year-old leaves were higher than those in 3-year-old leaves and leaf litters (Fig 3A and 3B). In contrast, the N/P ratio was lowest in 1-year-old leaves and the N/P ratio in 1-year-old leaves at Hunan was higher in LDCF than HDCF, while the N/P ratio in leaf litters at Fujian was lower in LDCF than HDCF (Fig 3C).

**Fig 4:**
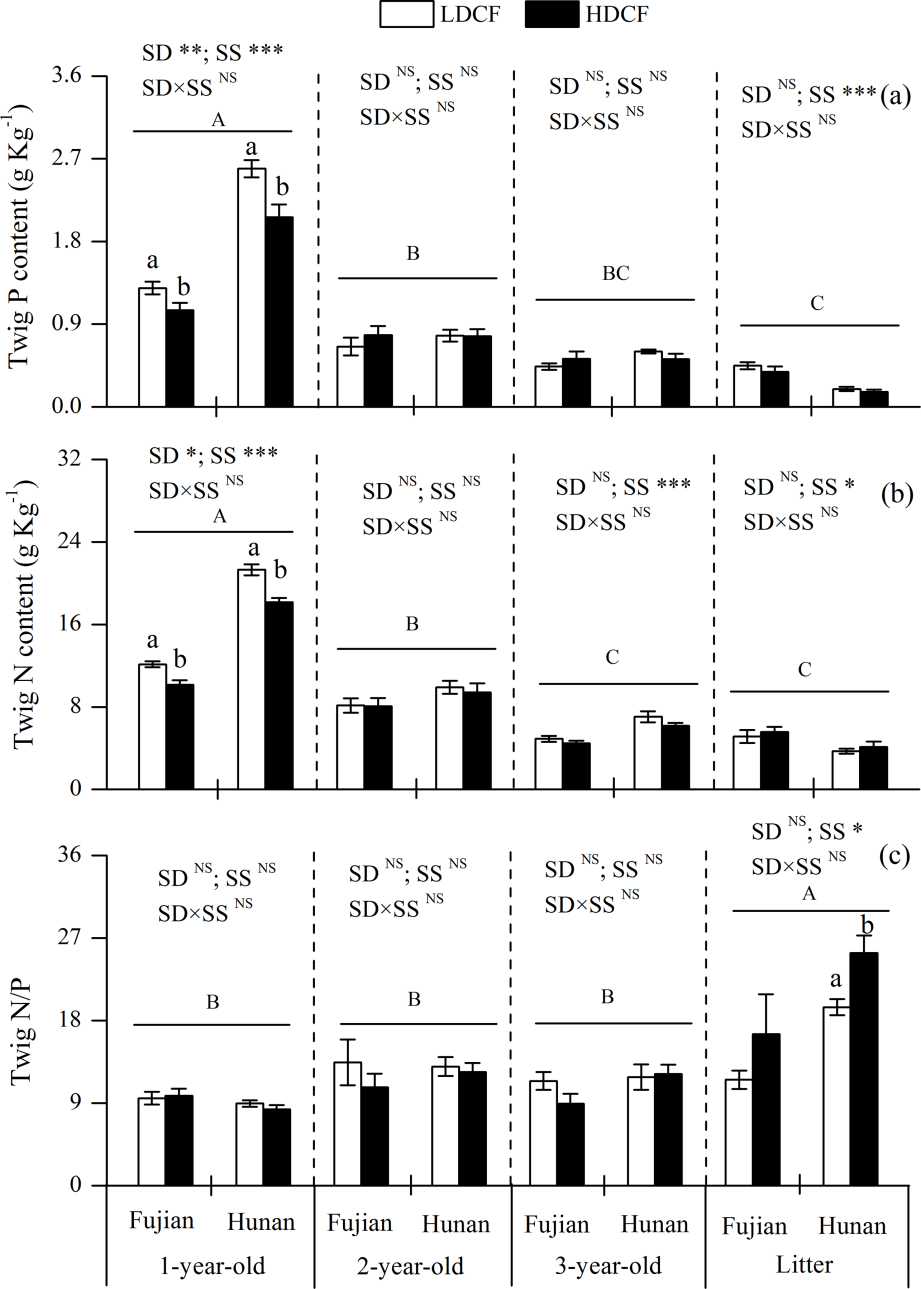
Nitrogen and phosphorus concentrations and their stoichiometry in twigs with different ages in low density and high density Chinese fir plantations (LDCF vs. HDCF) at two sites of subtropical China. Note: Mean±1 standard error. Different small letters indicate significant differences between LDCF and HDCF in twigs of the same age at the 0.05 level. Different capital letters indicate significant differences among twigs of different ages at the 0.05 level. SD: stand density. SS: study site. ^NS^ not significant, * *p* < 0.05, ** *p* < 0.01, *** *p* < 0.001.

Similarly, significant effects of stand density on twig nutrients were detected mainly in 1-year-old twigs. The P and N concentrations in 1-year-old twigs were higher in LDCF than HDCF, and were significantly higher than those in 2-year-old twigs, 3-year-old twigs and twig litters (Fig 4A and 4B). Meanwhile, N/P ratio in twig litters at Hunan was significantly lower in LDCF than HDCF (Fig 4C).

**Fig 5:**
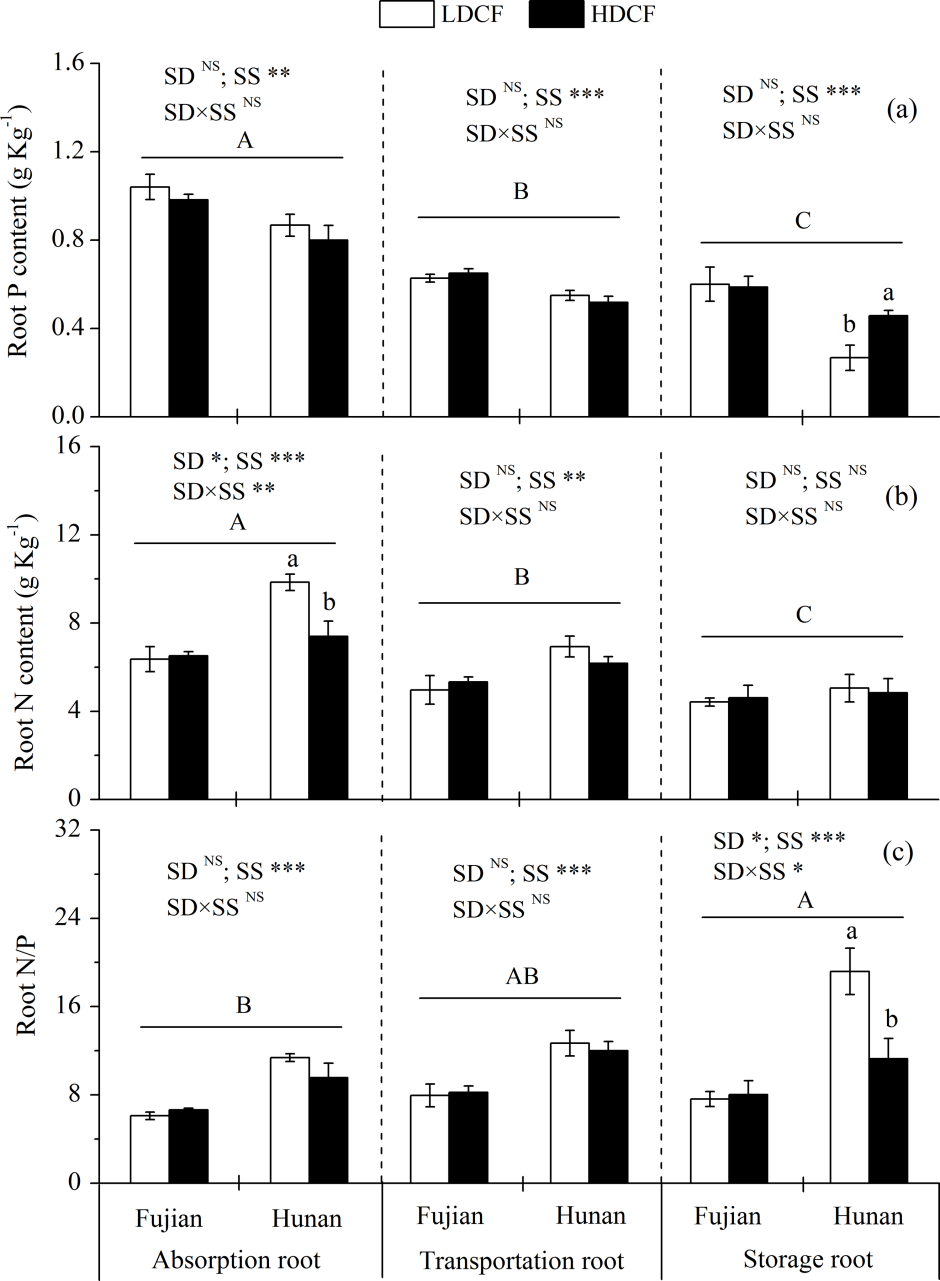
Nitrogen and phosphorus concentrations and their stoichiometry in various functional roots in low density and high density Chinese fir plantations (LDCF vs. HDCF) at two sites of subtropical China. Note: Mean±1 standard error. Different small letters indicate significant differences between LDCF and HDCF in roots of the same age at the 0.05 level. Different capital letters indicate significant differences among roots of different ages at the 0.05 level. SD: stand density. SS: study site. ^NS^ not significant, * *p* < 0.05, ** *p* < 0.01, *** *p* < 0.001.

The absorption root N concentration was higher in LDCF than HDCF, while the storage root P concentration had opposite tendency at Hunan. The average P concentrations in transportation and storage roots were lower by 36.4% and 48.2%, respectively and the average N concentrations were lower by 22.3% and 37.1% than those in absorption roots, respectively. However, average N/P ratios were higher in transportation and storage roots than that in absorption roots (Fig 5).

In addition, phosphorus concentrations in 1-year-old leaves and twigs were positively correlated with rhizosphere soil labile P, while were negatively correlated with rhizosphere soil inert P (S1 Table). In contrast, the N concentrations in 1-year-old leaves and twigs were positively correlated with rhizosphere soil slow P and soil labile P, respectively (S2 Table).

### Rhizosphere effect and nutrient resorption efficiency

Positive rhizosphere effects were found for all P fractions at two sites except for weathered mineral P at Fujian. The rhizosphere effects of labile P at two sites and occluded P at Fujian were greater in LDCF than HDCF (Table 3). The highest and lowest values of the rhizosphere effect were found for labile P (2.05) in LDCF and weathered mineral P (0.82) in LDCF at Fujian, respectively.

**Table 3:** Rhizosphere effects of soil phosphorus fractions in low density and high density Chinese fir plantations (LDCF vs. HDCF) at two sites of subtropical China.

	Available P	Labile P	Slow P	Occluded P	Weathered mineral P	Extractable P	Inert P
Fujian							
LDCF	1.09±0.04	2.05±0.40a	1.09±0.10	1.36±0.08a	0.82±0.16	1.16±0.10	1.02±0.04
HDCF	1.17±0.16	1.35±0.17b	1.06±0.04	1.11±0.04b	0.95±0.08	1.06±0.04	1.03±0.01
Hunan							
LDCF	1.06±0.20	2.28±0.11a	1.30±0.11	1.24±0.16	1.34±0.29	1.28±0.13	1.12±0.07
aHDCF	1.08±0.02	1.88±0.19b	1.23±0.13	1.13±0.20	1.55±0.25	1.35±0.17	1.21±0.08
Variance analysis of *F*-statistics^#^							
SD	0.18^NS^	5.69*	3.77^NS^	0.13^NS^	3.45^NS^	2.78^NS^	6.48*
SS	0.17^NS^	1.23^NS^	0.26^NS^	1.77^NS^	0.80^NS^	0.02^NS^	0.72^NS^
SD×SS	0.05^NS^	4.58*	0.03^NS^	0.27^NS^	0.34^NS^	0.44^NS^	0.57^NS^

Value = Mean±1 standard error. Different letters indicate significant differences between LDCF and HDCF at 0.05 level. SD: stand density. SS: study site.

^#^ Significance level of *F* values:

^NS^ not significant

* *p* < 0.05.

**Fig 6:**
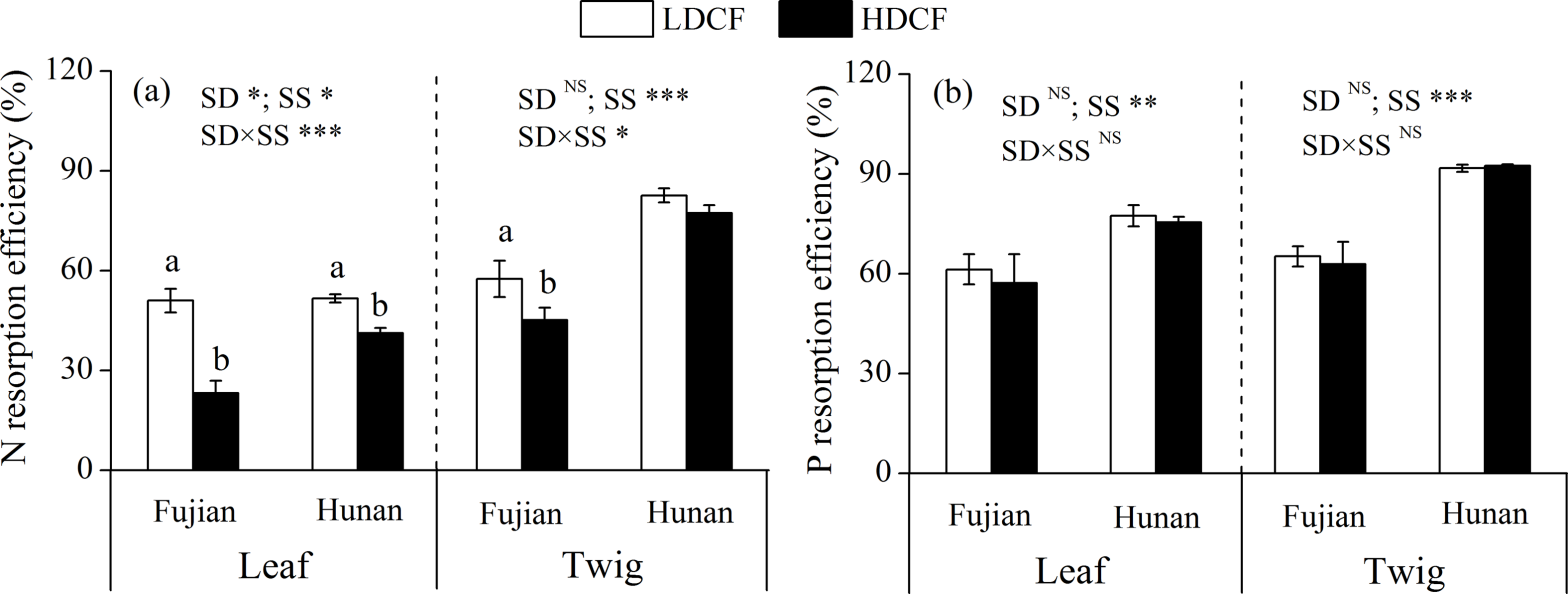
Nitrogen and phosphorus resorption efficiencies in leaves and twigs in low density and high density Chinese fir plantations (LDCF vs. HDCF) at two sites of subtropical China. Note: Mean±1 standard error. Different small letters indicate significant differences between LDCF and HDCF at the 0.05 level. SD: stand density. SS: study site. ^NS^ not significant, * *p* < 0.05, ** *p* < 0.01, *** *p* < 0.001.

Both leaf and twig N resorption efficiencies were higher in LDCF (average 46.1% and 67.4%, respectively) than HDCF (average 37.3% and 63.8%, respectively) and stand density had significant effects on leaf N resorption efficiency (Fig 6A). In contrast, leaf and twig P resorption efficiencies were not significantly different between LDCF and HDCF at two sites (Fig 6B). Average P resorption efficiencies in leaves (67.8%) and twigs (78.0%) were higher than the corresponding N resorption efficiencies (Fig 6).

## Discussion

### The effect of stand density on soil P supply

The stand density effects on forests are mainly associated with competition between trees for nutrients and environmental resources [6,32]. In many forest ecosystems, P is the key nutrient limiting plant growth, especially in subtropical red soil areas [10,11,26], where soil is rich in iron and aluminum because of the high degree of weathering, resulting in the adsorption and chemical fixation of P by conversion to Fe-P and Al-P [13,26]. In addition, high N deposition in subtropical areas enhances the plant’s demand for P [11]. In the current study, stand density had no significant effect on soil total P but had a significant effect on most soil active P fractions and extractable P. We found rhizosphere soil labile P, slow P, occluded P and extractable P were higher in LDCF than HDCF at two study sites (Figs 1 and 2). A large number of active P fractions in LDCF could be enhanced by the strong positive rhizosphere effect (Table 3), since previous studies have reported that low density forest usually has higher light penetration and photosynthetic efficiency resulting in high growth efficiency and high underground carbon allocation [3,7]. In contrast, high density forest has lower productivity due to competition between trees resulting relative lack of water and nutrients in the soil. For instance, Blevin et al. (2005) reported that a decrease in stand density (thinning) increased plant leaf biomass and tree DBH in a 36-year-old lodgepole pine (*Pinus contorta*) forest [3]. Similarly, López et al. (2003) found that fine biomass increased by more than 100% and production increased by 76% after thinning in a *Quercus ilex* forest [33]. Moreover, the magnitude of rhizosphere effects on P availability in forest soils was coupled to belowground carbon allocation patterns and rhizosphere carbon fluxes, given that a large proportion of the P was bound to organic matter through ester bonds, and roots release phosphatase enzymes and low-molecular-weight organic acids to access P [34,35]. Therefore, we speculate that the high plant productivity (such as DBH) in LDCF might increase the allocation of photosynthetic products to belowground tissues (such as roots) and increase root exudates to soil, which enhances the rhizosphere effect and promotes the activation of P fractions.

The Hedley fractionation addresses the disadvantages of traditional methods of P determination, since traditional methods can not distinguish the inorganic P and organic P together and are unable to link the P fractions to plant-availability [12,15]. Usually, P is easily fixed in the soil and the root P concentration is much higher than that in rhizosphere soil, which leads to the result that plant P absorption is difficult. However, plants are able to enhance the soil P uptake by mycorrhiza [36]. The mycorrhiza of Chinese fir is vesicular arbuscular mycorrhiza and the mycelium of arbuscular mycorrhizal fungi (AMF) has the trait of absorbing soil inorganic P forms, which greatly extends the range of plant P absorption. Moreover, the infection of AMF often causes a series of changes in soil physical and chemical properties promoting the mineralization of organic P and the dissociation of insoluble P. In addition, the organic acids secreted by AMF may decrease soil pH and increase the activation of soil P [37]. Therefore, available P and labile P are inorganic forms of P, which can be directly absorbed by Chinese fir plants [18]. In the current study, LDCF had higher bulk soil available P and rhizosphere soil labile P concentrations than HDCF at two sites, indicating a higher active P supply capacity in LDCF. Slow P was extracted with NaOH solution and contained organic and inorganic forms adsorbed by soil iron, aluminum and clay. Similarly, occluded P was mainly present in the surface of soil aggregates and was extracted after ultrasonic dispersion [14]. Although the slow P and occluded P can not be quickly absorbed by plants, these forms provide a plant-available P pool, since soil P forms can be transformed into each other [16,17]. Similarly, extractable P was defined as the sum of extracted P fractions. It represents the potential active P pool for using by plants and microorganisms after the soil environment changes [15] and was higher in LDCF than in HDCF soils at two sites. Thus, the decrease of stand density not only had a high soil available P concentration, but also improved the overall P supply capacity.

### The effect of stand density on tree tissue P and N distributions

Consistent with our hypothesis, 1-year-old leaf and twig P and N concentrations were higher in LDCF than that in HDCF, whereas no significant differences were found between two stand density plantations for P and N concentrations in 2-year-old and 3-year-old leaves and twigs or in transportation and stroage roots (Figs 3–5). Plant tissue nutrient concentration is often determined by soil nutrient supply [11,38]. In this study, the positive correlation between rhizosphere soil active P fractions, such as labile P, slow P and extractable P, and 1-year-old leaf P and N concentrations (S1 and S2 Tables) illustrated that enhanced soil active P supply could increase the tree tissue nutrient concentrations [38,39]. Moreover, 1-year-old leaves and twigs are young tissues, with relatively high nutrient demand, and are thus susceptible to soil nutrient contents. This is consistent with the results of Chen *et al*. (2015), who reported that N and P additions increased soil nitrate-N, ammonium-N and available P concentrations and the response of N and P additions mainly occurred in the new foliage and twigs in Chinese fir plantations. Finally, we found the P and N concentrations in leaves and twigs generally decreased with increasing tissue age. A possible mechanism is organic P and N hydrolysis as plant organs senesce, leading to the recycling of hydrolyzate amino acids and inorganic P to living organs. It had been reported that protein hydrolyzed and subsequently retranslocated as amino acids was equivalent to 82–91% of the N removed from senescing leaves [40,41].

The N/P ratio has successfully been used to describe nutrient limitation on plant growth. Braakhekke and Hooftman (1999) considered that when plant leaf N/P>14 and leaf total P concentrations are less than 1 g kg^−1^, then the ecosystem is limited by P and when the leaf N/P<10 and N concentrations are less than 20 g kg^−1^, the ecological system is N limited [42]. However, Koerselman and Meuleman (1996) suggested that an N/P>16 indicates P limitation on a community level, while an N/P<14 is indicative of N limitation and an N/P between 14 and 16 indicates that either N or P can be limiting or plant growth is colimited by N and P together [43]. Although the ecological stoichiometry for N and P limitation was different, it was clear that leaf P limitation enhanced with increasing age and the Chinese fir plantation of the current study was primarily P limited. This is because the leaf N/P ratio significantly increased with age and the average N/P ratios of 2-year-old, 3-year-old leaves and leaf litters were greater than 16 (18.2, 16.5 and 19.7, respectively) [42,43]. These results may be explained by the low concentrations and low percentages of plant-available soil P fractions in our study area.

### Responses of P rhizosphere effect and nutrient resorption efficiency to stand density

The rhizosphere effect and nutrient resorption are two important strategies of the plant nutrients acquisition, especially in poor nutrient conditions [21,23]. Plants usually adjust nutrient capture strategies to obtain more nutrients for growth in infertile soils [24,25,40]. Contrary to our hypotheses, either the rhizosphere effect of the most soil active P forms or the leaf and twig N resorption efficiencies were lower in HDCF than LDCF, which may be attributed to the differences of carbohydrate synthesis and distributions in the two different Chinese fir plantation densities. The increase of rhizosphere effect and nutrient resorption requires energy provided by carbohydrates, whereas light penetration and single tree leaf biomass may be lower in HDCF than in LDCF, resulting in low photosynthetic efficiency and primary productivity in HDCF [3,7]. Although we did not directly measure the primary productivity and biomass partitioning, it had reported the effects of stand density on these variables [44,45]. Analysis of 10-year-old Manchurian Ash (*Fraxinus mandshurica*) plantations indicated that the mean single tree biomass and root biomass increased with the decrease of initial planting density [46]. Similarly, Bormann and Gordon (1984) found trees in low density stands had the highest mass, volume and surface-area components in 5-year-old red alder (*Alnus rubra*) plantations with different stand densities [45]. Therefore, we speculate that the higher primary productivity and root biomass may result in the increasing carbohydrate allocation for rhizosphere process and nutrient resorption in the low density stands.

Moreover, recent studies have indicated that plant nutrient concentrations and resorption are highly dependent on soil nutrient status [24,25]. In this study, the Chinese fir plantation of two stand density had low percentage of extractable P on total P (mean 14.7%) and plant-available P (mainly contains available P and labile P) only accounting for 1.55% in LDCF and 1.54% in HDCF of their total P. This suggests that the lack of plant-available P might be the main reason for the higher P resorption efficiencies in leaves and twigs [10,11,26]. In addition, the leaf and twig N/P generally increased with increasing age, indicating a gradually increased plant P deficiency with growth, which may induce positive rhizosphere effects of most P fractions and extractable P in LDCF and HDCF.

On an overall basis, our results revealed that thinning may be helpful to increase soil active P supplies and meet tree nutrient requirements through the comparation of P and N concentrations in plant rapid turnover organs and soils between low and high density Chinese fir plantations. However, the stand density on soil-tree nutrient cycling in Chinese fir need be further concerned since only 2 stand densities were compared, and soil microbial properties and plant physiological traits were not observed in this study.

## Conclusions

Stand density was a key factor to affect nutrient internal cycles in subtropical Chinese fir plantation forests. Compared with HDCF, LDCF showed a higher P availability in rhizosphere soil and a higher P and N concentrations in young leaf and twig tissues. Leaf N/P ratio increased with age, and the average N/P in 2-year-old and 3-year-old fresh leaves and in leaf litters were all greater than 16. Both leaf and twig P resorption efficiencies were higher than their N resorption efficiencies. Our results suggested that the Chinese fir plantation would be a P-limited ecosystem and moderate thinning might help to improve the P internal cycling.

## Supporting information

S1 Table The coefficients of Pearson’s correlations between rhizosphere soil phosphorus fractions and tree tissue phosphorus concentrations in Chinese fir plantation of subtropical China. * *p*<0.05, ** *p*<0.01.
(DOC)

S2 Table The coefficients of Pearson’s correlations between rhizosphere soil phosphorus fractions and tree tissue nitrogen concentrations in Chinese fir plantation of subtropical China. * *p*<0.05, ** *p*<0.01, *** *p*<0.001.
(DOC)
