# Supplementary material for: Soil phosphorus functional fractions and tree tissue nutrient concentrations influenced by stand density in subtropical Chinese fir plantation forests
Source: PLoS One. 2017 Oct 26;12(10):e0186905. doi: 10.1371/journal.pone.0186905 (PMC5658083; doi:10.1371/journal.pone.0186905)
Supplement: S1 Table — * p<0.05, ** p<0.01. (DOC) [file pone.0186905.s001.doc]

**S1 Table. The coefficients of Pearson’s correlations between rhizosphere soil phosphorus fractions and tree tissue phosphorus concentrations in Chinese fir plantation of subtropical China.**

| Variables | Available P | Labile P | Slow P | Occluded P | Weathered mineral P | Extractable P | Inert P |
| --- | --- | --- | --- | --- | --- | --- | --- |
| Leaf | | | | | | | |
| 1-year-old | −0.30 | 0.66** | 0.04 | -0.25 | -0.48 | 0.03 | -0.52* |
| 2-year-old | 0.14 | 0.03 | −0.05 | -0.05 | 0.22 | -0.27 | 0.08 |
| 3-year-old | −0.23 | 0.12 | 0.05 | -0.19 | -0.30 | -0.08 | -0.04 |
| Twig | | | | | | | |
| 1-year-old | −0.32 | 0.57* | −0.02 | -0.49 | -0.43 | -0.12 | -0.71** |
| 2-year-old | −0.36 | 0.14 | 0.09 | -0.40 | -0.32 | -0.18 | -0.15 |
| 3-year-old | −0.50* | 0.33 | −0.21 | -0.55* | -0.29 | -0.48 | -0.30 |
| Root | | | | | | | |
| Absorption | 0.32 | −0.42 | 0.28 | 0.68** | 0.42 | 0.44 | 0.66** |
| Transportation | 0.44 | −0.33 | 0.17 | 0.54* | 0.64** | 0.17 | 0.70** |
| Storage | 0.41 | −0.21 | 0.14 | 0.56* | 0.15 | 0.17 | 0.37 |

* *p*<0.05, ** *p*<0.01.
