# Supplementary material for: Soil phosphorus functional fractions and tree tissue nutrient concentrations influenced by stand density in subtropical Chinese fir plantation forests
Source: PLoS One. 2017 Oct 26;12(10):e0186905. doi: 10.1371/journal.pone.0186905 (PMC5658083; doi:10.1371/journal.pone.0186905)
Supplement: S2 Table — * p<0.05, ** p<0.01, *** p<0.001. (DOC) [file pone.0186905.s002.doc]

**S2 Table. The coefficients of Pearson’s correlations between rhizosphere soil phosphorus fractions and tree tissue nitrogen concentrations in Chinese fir plantation of subtropical China.**

| Variables | Available P | Labile P | Slow P | Occluded P | Weathered mineral P | Extractable P | Inert P |
| --- | --- | --- | --- | --- | --- | --- | --- |
| Leaf |  |  |  |  |  |  |  |
| 1-year-old | 0.32 | 0.38 | 0.49* | 0.40 | −0.31 | 0.52* | −0.06 |
| 2-year-old | 0.11 | 0.12 | 0.13 | 0.26 | 0.38 | 0.02 | 0.31 |
| 3-year-old | −0.11 | 0.59* | 0.26 | −0.07 | −0.38 | 0.22 | −0.20 |
| Twig |  |  |  |  |  |  |  |
| 1-year-old | −0.30 | 0.50* | −0.13 | −0.61* | −0.54* | −0.19 | −0.82*** |
| 2-year-old | −0.17 | 0.48 | 0.12 | −0.44 | −0.34 | −0.06 | −0.33 |
| 3-year-old | −0.11 | 0.48 | 0.21 | −0.46 | −0.50* | −0.10 | −0.62** |
| Root |  |  |  |  |  |  |  |
| Absorption | −0.18 | 0.52* | 0.12 | −0.52* | −0.44 | −0.08 | −0.55* |
| Transportation | −0.35 | 0.50* | 0.14 | −0.63** | −0.54* | −0.10 | −0.58* |
| Storage | −0.41 | −0.01 | −0.26 | −0.20 | −0.22 | −0.24 | −0.19 |

* *p*<0.05, ** *p*<0.01, *** *p*<0.001.
